# EHMTI-0388. Levels of calcitonin-gene related peptide in medication overuse headache – a pilot study

**DOI:** 10.1186/1129-2377-15-S1-I9

**Published:** 2014-09-18

**Authors:** SB Munksgaard, E Frandsen, L Bendtsen, RH Jensen

**Affiliations:** 1 Danish Headache Center Department of Neurology, Glostrup Hospital University of Copenhagen, Glostrup, Denmark; 2 Department of Diagnostics Clinical Physiology and Nuclear Medicine, Glostrup Hospital, Glostrup, Denmark

## Background

Medication-overuse headache (MOH) is a chronic, secondary headache caused by excessive intake of symptomatic medications for an underlying headache, most often tension-type headache or migraine. Previously, elevated calcitonin-gene-related peptide (CGRP) levels were found in migraine patients increasing with migraine frequency and in animal MOH models.

## Aim

To test whether MOH patients have increased CGRP levels before withdrawal therapy compared with healthy volunteers, and further, if the CGRP level would normalize after withdrawal in parallel with the headache-frequency decrease.

## Methods

Blood samples from 16 MOH patients before and 6 months after withdrawal start and from 30 healthy volunteers of matching sex and age were analyzed. CGRP concentrations were determined by radioimmunoassay using antibody AB4-2905 and α-CGRP as calibrator. Free and antibody-bound tracer were separated by Sac-Cel separation. The analyses were done blinded to subject group.

## Results

Median CGRP concentration was 44.4 pmol/L in MOH patients before withdrawal and 43.8 pmol/L in healthy volunteers (p=0.72). After withdrawal, median CGRP-concentration reduction was 2.1% (p=0.76). Median headache-frequency reduction was 42.5%; from 25 days/month to 12 days/month. Female patients tended to have higher CGRP levels than male patients; 50.6 vs 39.6 pmol/L (p=0.38). We were not able to detect any differences in CGRP concentration according to type of drug overused, in relation to underlying headache type, or to reduction in headache frequency.

## Conclusions

No change in CGRP was detected despite dramatic reduction in headache frequency after detoxification of MOH patients. Thus, our results do not support the notion that CGRP is involved in MOH.

No conflict of interest.

